# Benefits and Risks of Medications Used in the Management of Hypotension: A Review

**DOI:** 10.7759/cureus.51608

**Published:** 2024-01-03

**Authors:** Thangwaritorn Skylynn, Thomas Abel, Lee Christopher, Ghafary Suliman, Rivera Dominic, Varughese Joel, Zeyu Yu, Sudhakar Pemminati

**Affiliations:** 1 Department of Biomedical Education, California Health Sciences University College of Osteopathic Medicine, Clovis, USA; 2 Department of Pharmacology, California Health Sciences University College of Osteopathic Medicine, Clovis, USA

**Keywords:** adrenergic medications, norepinephrine reuptake inhibitors, autonomic control, cardiovascular, hypotension

## Abstract

This comprehensive literature review addresses the scarcity and limited study of hypotension treatments compared to abundant antihypertensive drugs. Hypotension, categorized as absolute, relative, or orthostatic, has diverse causes. This review explores various treatments, including drugs affecting the sympathetic nervous system, such as midodrine, dihydroergotamine, and ergotamine, which have shown efficacy in managing hypotension. Dopamine agonists/antagonists and other drugs such as ephedrine, norepinephrine, and fludrocortisone are also discussed, each with distinct mechanisms and applications. Additionally, adjunctive agents such as non-steroidal anti-inflammatory agents, caffeine, and monoamine oxidase inhibitors are reviewed for their effects on blood pressure. This review underscores the importance of understanding the efficacy and safety profiles of hypotension treatments to guide healthcare professionals in optimal drug selection and management, emphasizing the need for further research and comparative studies for evidence-based guidelines.

## Introduction and background

**Figure 1:**
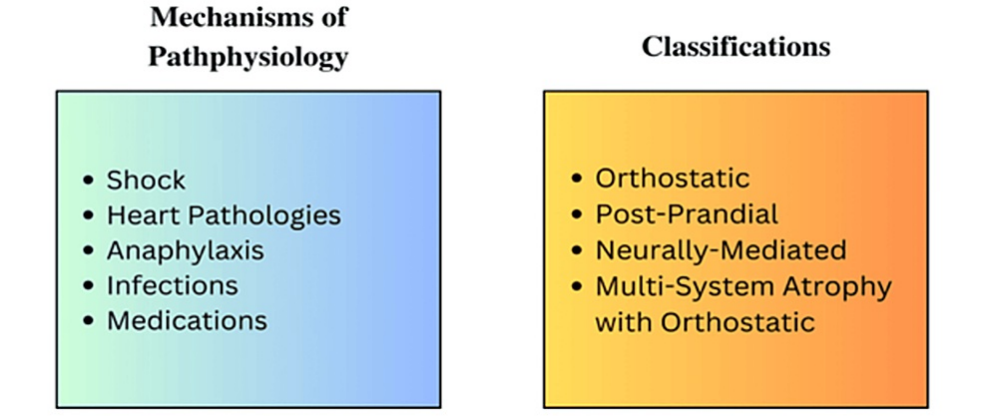
Causes and classification of hypotension. Image credits: Ghafary Suliman.

The definition of hypotension can vary but is commonly accepted as a decrease in blood pressure, typically identified as values lower than 90/60 mmHg [1]. Hypotension can also be identified by utilizing mean arterial pressure, with pressures less than 65 mmHg considered hypotensive [1]. The maintenance of blood pressure is controlled by the ability of the sympathetic nervous system to increase blood pressure through the constriction of arterioles and an increase in heart rate, as well as the ability of the parasympathetic nervous system to lower blood pressure [1]. There are four major classifications for hypotension, which include orthostatic hypotension, postprandial hypotension, neurally mediated hypotension, and multiple system atrophy with orthostatic hypotension [2]. Various bodily mechanisms can lead to a hypotensive state, including shock, heart pathologies, anaphylaxis, infections, and side effects of medications [2], as depicted in Figure 1. Given the various human conditions and pathologies, many drugs in today’s medical field are used to combat hypotension, and each available drug has a unique mechanism of action that may make one more suitable for a specific hypotensive condition.

## Review

Drugs affecting the sympathetic nervous system to manage hypotension

Midodrine

Midodrine is a Food and Drug (FDA)-approved drug used to treat orthostatic hypotension. It functions as an α-1 agonist by binding to alpha-adrenergic receptors, stimulating the constriction of arteries and veins [3]. Midodrine has been reported to increase standing systolic blood pressure, reduce lightheadedness symptoms, and improve global symptom relief scores for both patients and investigators [4]. Additionally, midodrine has been reported to alleviate symptoms of intradialytic hypotension [3]. The most common adverse effects in patients taking midodrine include pilomotor reactions, urinary retention, and supine hypertension [4]. Hypotensive patients with spinal cord injury receiving midodrine 10 mg have significantly elevated blood pressure and decreased episodes of hypotension [5].

Ergotamine

Ergotamine is commonly used to treat migraines due to its vasoactive properties. Ergotamine tartrate has a structural similarity to norepinephrine, epinephrine, dopamine, and serotonin, allowing it to act as an α-1 receptor agonist and a serotonin receptor agonist [6]. These vasoactive properties of ergotamine can also be used to treat hypotension. However, the oral bioavailability of ergotamine is less than 1%, which limits its effectiveness as a treatment for hypotension [6]. To improve its effectiveness and oral absorption rate, ergotamine is often combined with caffeine. A study reported that the combination of ergotamine and caffeine increased systolic blood pressure and improved presyncopal symptoms [7].

Yohimbine

Yohimbine is an α-2 receptor antagonist and, as a result, it increases plasma epinephrine and norepinephrine concentrations, which, in turn, raise heart rate, systolic blood pressure, and local blood flow [8]. These effects of yohimbine can make it a useful treatment for hypotension. In particular, yohimbine is used to treat orthostatic hypotension induced by the tricyclic antidepressant clomipramine. A study reports that patients suffering from clomipramine-induced hypotension and treated with yohimbine experienced a significant increase in systolic pressure when in an upright position, as well as an improvement in dizziness [9]. Another study reported that yohimbine also increased standing diastolic blood pressure and presyncope symptoms in patients with neurogenic orthostatic hypotension [10]. However, yohimbine has limited availability in the United States and is only marketed as a dietary supplement. Consequently, yohimbine is not typically used to treat hypotension in the United States.

Droxidopa

Droxidopa is an FDA-approved drug used to treat neurogenic orthostatic hypotension (nOH). nOH is caused by a decrease in the production of norepinephrine due to postganglionic sympathetic neuron degeneration and, as a result, deficient vascular adrenoceptor activation. Sympathetic neuron degeneration is commonly observed in conditions such as Parkinson’s disease, pure autonomic failure, or multiple system atrophy. Droxidopa can compensate for the decrease in norepinephrine because it is converted into norepinephrine by the aromatic amino acid decarboxylase, an enzyme abundantly expressed throughout the body. Studies have reported that droxidopa increases systolic blood pressure and improves symptoms of orthostatic hypotension. Droxidopa is generally well tolerated, but its side effects include nausea, dizziness, vision disturbances, weakness, and fatigue [11].

Dobutamine

Dobutamine is currently on the market as a short-term FDA-approved drug that affects the β adrenoceptors of the heart. It is mainly prescribed for the treatment of cardiogenic shock, heart failure, and in those with low blood pressure from decreased cardiac output. In one drug trial of dobutamine versus milrinone, it was found that higher outpatient hospital mortality risks were associated with dobutamine. Additionally, because of its cardiac effects dobutamine might cause worsening of tachycardia symptoms, introduce heart arrhythmias, and increase the myocardial oxygen demand [12]. However, it should be noted that the study did not directly measure hypotension outcomes, but rather mortality as the primary outcome which could have been influenced by other medications, changes in renal function, and other confounding influences. Other studies of dobutamine versus milrinone seem to contradict these findings and showed milrinone was associated with longer hospital stays, more intensive care unit admissions, and increased necessity for renal replacement therapy, but no significant difference in mortality outcomes between the two groups [13]. Additional studies have shown that it may be effective in the treatment of hypotensive preterm neonates as it improves mean arterial pressure, superior vena cava blood flow, and higher right ventricular outputs in comparison to treatments of dopamine [14,15].

Ephedrine

Ephedrine is both an alpha and beta-receptor agonistic activator. It releases norepinephrine by indirect activation and leads to α-1 and β-1 effects [16], with β-1 increasing the heart rate and α-1 causing peripheral vasoconstriction. Ephedrine is indicated for use in patients for prophylaxis and in cases of maternal hypotension during labor. In a sample of 80 full-term parturients, those who were given ephedrine via rapid intravenous bolus, compared to the placebo, showed a significant increase in heart rate and blood pressure [17]. Additionally, 150 patients undergoing valve surgery who were administered 0.07 to 0.1 mg/kg of ephedrine before propofol anesthesia showed improvements in mean arterial pressure, systemic vascular resistance index, cardiac index, stroke volume index, and left ventricular stroke work index [18]. Dusitkasem et al. showed that ephedrine does have an increased risk for fetal tachycardia and acidosis [19].

Norepinephrine

Norepinephrine is a potent vasoconstrictor and a precursor to epinephrine. Its main action is to increase systemic vascular resistance and heart rate. Norepinephrine is commonly indicated for use in cases of shock and hypotension [16]. Hasanin et al. compared norepinephrine to phenylephrine in a double-blinded controlled trial and showed that norepinephrine effectively maintained maternal systolic blood pressure [20]. While the study did show favorable outcomes for reduced post-spinal epidural hypotension, there were adverse effects such as nausea, vomiting, bradycardia, and dry gangrene.

Epinephrine is both a strong alpha-adrenoceptor and beta-adrenoceptor agonist [21]. It is another potent vasoconstrictor. Beta effects are shown at lower levels to increase both cardiac output and heart rate compared to α-1, which, at higher levels, is shown to increase both afterload and mean arterial pressure [16]. Wang et al. compared the effectiveness of epinephrine to phenylephrine during cesarean delivery, which demonstrated that maternal hypotension, bradycardia, nausea, and vomiting were similar in both groups, with the incidence of maternal bradycardia being reduced in the epinephrine group [21]. The study also concluded that there was a greater umbilical artery pH in the epinephrine group, suggesting that it is associated with better uteroplacental perfusion.

Etilefrine

Etilefrine is a well-known α-1 adrenergic receptor agonist and is known to be a potent vasoconstrictor, increasing blood pressure. Etilefrine is useful in the treatment of orthostatic hypotension induced by clozapine. Etilefrine therapy showed a decrease in the orthostatic reaction. Adverse effects include nausea, tremors, and palpitations [22].

Dopamine

Dopamine is synthesized via the same sequence as norepinephrine and is a precursor to norepinephrine. Dopamine works in a dosage-dependent fashion. At low infusion rates, it binds to alpha and beta receptors, causing vasodilation. At intermediate doses, it stimulates beta receptors, increasing myocardial contractility, SA rate, and impulse conduction in the heart. At high rates, dopamine affects alpha receptors, causing vasoconstriction and a rise in blood pressure. Therefore, dopamine is utilized to treat hemodynamic imbalances in shock [23-25]. The findings are summarized in Table 1.

**Table 1:** Drugs that affect the sympathetic nervous system. HT: hydroxytryptamine; CNS: central nervous system

Medication, author, year, and country	Study population	Mechanism of action	Benefits	Adverse effects/Other comments
Midodrine: Low et al., 1997, United States [4]	171	α-1 receptor agonist	Increases standing systolic blood pressure and alleviates intradialytic hypotension	Pilomotor reactions, urinary retention, supine hypertension, scalp paraesthesias, and heartburn
Ergotamine: Arnold et al., 2014, United States [7]	12	α-1 receptor agonist 5HT receptor agonist	Increases systolic blood pressure and improves presyncope symptoms	Nausea, headache, malaise, vomiting. Contraindicated in patients with coronary or peripheral artery disease
Yohimbine: Lecrubier et al., 1981, France [9]	11	α-2 receptor antagonist	Treats clomipramine-induced hypotension	Nervousness, insomnia, anxiety, and increased urinary frequency
Droxidopa: Kaufmann et al., 2014, United States [11]	162	Increases norepinephrine concentration	Increases systolic blood pressure and improves neurologic orthostatic hypotension symptoms	Nausea, dizziness, vision disturbance, weakness, and fatigue
Dobutamine: Zhu et al., 2021, China [13]	235	β-adrenoceptor agonist	Less cardiac arrhythmia occurrence, shorter hospital stays, and less requirement of renal replacement therapy in comparison to milrinone	Cardiotoxic and might induce cardiomyopathy
Ephedrine: Biricik et al., 2021, Turkey [16]	Electronic literature review	α and β receptor agonist. This leads to the activation of β-1, which increases heart rate	Used in the treatment of prophylaxis and maternal hypotension	Palpitations, headaches, dizziness, nausea, and vomiting. Contraindicated in patients with acute hypertension/tachycardia
Norepinephrine: Biricik et al., 2021, Turkey [16]	Electronic literature review	Stimulates α-1 receptors	Used for treatment of type 1 anaphylactic reactions	Tachycardia, hypertension, headache, and anxiety
Epinephrine: Hasanin et al., 2023, Egypt [20]	271	Acts on α-1 receptors	Used for the treatment of type 1 anaphylactic reactions	Tachycardia, hypertension, headache, and anxiety
Etilefrine: Tanzer et al., 2022, Australia [22]	Electronic literature review	α-agonist with a potent vasoconstrictor	Increases blood pressure and reduces symptoms of hypotension such as dizziness and headaches	CNS effects, difficulty with micturition
Dopamine: Li et al., 2020, Taiwan [23]	7,410	α-receptor- and β-receptor-stimulating actions	Naturally occurring potent vasoconstrictor and an inotropic agent	Greater mortality has a higher incidence of arrhythmic events than that of norepinephrine and alters hypothalamic-pituitary function. Decreases prolactin and growth hormone levels, pulmonary vasodilator. Vasoconstrictive effects are preserved during hypoxia and severe acidosis

Norepinephrine-dopamine reuptake inhibitors

Atomoxetine

Atomoxetine is a selective norepinephrine reuptake inhibitor. It increases norepinephrine levels peripherally, and it dramatically raises blood pressure at small doses, specifically at doses of 18 mg. This medication can be useful in patients with severe symptoms of orthostatic hypotension, and it has been proven to be safe and effective, even in the elderly population. The side effects of atomoxetine include an increased heart rate and a significant increase in seated and standing systolic blood pressure when compared with placebo and current standards of care, such as midodrine. It has also been shown to raise systolic blood pressure only in patients with an intact central nervous system to avoid hypertension in patients treated with this drug [26]. The findings are summarized in Table 2.

**Table 2:** Norepinephrine-dopamine reuptake inhibitors.

Article name, author, year, country	Study population	Mechanism of action	Benefits	Adverse effects/Other comments
Atomoxetine Patel et al., 2018, United States [26]	Electronic literature review limited to human studies from January 2000 to May 2017	Increases norepinephrine levels and raises blood pressure with small doses	Improves orthostatic hypotension	Side effects include increased heart rate. Patients must have an intact central nervous system to avoid hypertension

Other classes of targets for treating hypotension

Fludrocortisone

Fludrocortisone is a synthetic mineralocorticoid and is commonly used to treat hypotension. As a mineralocorticoid, fludrocortisone increases the rate of sodium reabsorption and water retention [27]. Studies have shown that fludrocortisone increases systolic blood pressure, improves symptoms, and decreases orthostatic tachycardia in patients suffering from neurogenic orthostatic hypotension caused by diabetic neuropathy or Parkinson’s disease [28,29]. The most common adverse effects include supine hypertension, peripheral edema, and headaches [27,29]. The use of fludrocortisone is contraindicated in treating patients with hypertension, hyperalbuminemia, and systemic fungal infections [27].

Pyridostigmine

Pyridostigmine belongs to a class of cholinesterase inhibitors and works to increase levels of acetylcholine, a neurotransmitter involved in muscle movement. Typically used in the treatment of myasthenia gravis, pyridostigmine may be prescribed off-label or in clinical trials for other conditions such as hypotension. In a comparison trial of pyridostigmine bromide versus fludrocortisone, the latter seemed to be more effective in raising mean arterial blood pressure and peripheral systolic supine blood pressure. Side effects of pyridostigmine bromide included softening of stool, lowered sodium levels, increased heart rate on Schellong maneuver, dizziness, and dry mouth [28]. Compared to midodrine, pyridostigmine also induced supine systolic blood pressure. The most effective treatment for hypotension appeared to be a combination trial of both midodrine and pyridostigmine to improve both systolic and diastolic blood pressure. Researchers noted side effects such as headaches, dizziness, gastrointestinal upsets, limb tremors, and potentially depression, lethargy, and sleep disturbances [30,31]. Similarly, the combination of pyridostigmine with other medications such as atomoxetine and propranolol or bisoprolol proved to be more efficacious than solely pyridostigmine [32,33].

Octreotide

Octreotide is a synthetic peptide that acts as a somatostatin analog, mimicking the action of somatostatin. Primarily used in other conditions such as acromegaly, gastroenteropancreatic neuroendocrine tumors, esophageal varices, or secretory diarrhea, octreotide may also be used off-label for postprandial hypotension. Octreotide improved fasting postural hypotension, fatigue, dizziness, standing heart rate, orthostatic syncope, increased stroke distance, cardiac index, resting skin temperature, systemic vascular resistance, and lowered plasma insulin levels but did not improve exercise-induced hypotension. It should be noted, however, that the effects of octreotide may be transient, with the first injection treatment having the most efficacy. Octreotide was well tolerated with no effects on nausea, abdominal cramps, or pain, though caution should be exercised for those with liver cirrhosis [34-36]. It is also known to increase supine hypertension, with researchers recommending the choice of other hypotensive therapies such as midodrine, mestinon, and subcutaneous octreotide instead of the very expensive intravenous octreotide [37].

Acarbose

Acarbose is an α-glucosidase inhibitor. It is largely used to treat type 2 diabetes because it slows down the small intestine’s enzymatic breakdown of carbohydrates. This enables more efficient metabolization of absorbed glucose. Acarbose may also be used to treat postprandial hypotension (PPH) in patients with severe autonomic failure because of its ability to effectively attenuate the drop in blood pressure that occurs after meals. Treatment with acarbose can cause a decrease in plasma insulin levels via lowering plasma glucose levels. As insulin is a recognized vasodilator, lowering its plasma levels reduces PPH [38]. Acarbose has a similar safety profile to placebo. Very rare instances of reversible liver transaminase elevations have been reported [39]. The findings are summarized in Table 3.

**Table 3:** Other classes of targets for treating hypotension.

Medication, author, year, country	Study population	Mechanism of action	Benefits	Adverse effects/Other comments
Fludrocortisone: Campbell et al., 1976, United Kingdom [29]	14	Increases sodium reabsorption and water retention	Increases systolic blood pressure	Use caution in patients with congestive cardiac failure or nephrotic syndrome
Pyridostigmine: Byuan et al., 2017, South Korea [30]	87	Cholinesterase inhibitor	Improves orthostatic blood pressure drops and increases supine systolic blood pressure	Aggravated dizziness, headache, gastrointestinal upsets (nausea and diarrhea), limb tremors, and visual disturbances. Can potentially cause depression, lethargy, and sleep disturbances
Octreotide: Smith et al., 1995, United Kingdom [34]	18	Somatostatin analog	Improves fasting postural hypotension before exercise, blood pressure quicker to recover to pre-exercise levels, increases heart stroke distance, increases heart cardiac index, increases resting skin temperature, and lowers plasma insulin levels	Does not seem to reduce exercise-induced hypotension
Acarbose: Shibao et al., 2007, United States [38]	13	α-glucosidase inhibitor	Treats type 2 diabetes by slowing down the breakdown of carbohydrates	Similar safety profile to placebo. Very rare instances of elevated liver transaminases

Adjunctive therapy

Non-steroidal Anti-inflammatory Agents

The primary mode of action for non-steroidal anti-inflammatory drugs (NSAIDs) involves blocking the action of the enzyme cyclooxygenase, essential for the formation of eicosanoids, leading to the therapeutic effects attributed to the reduced levels of these eicosanoids [40]. The FDA approved as antipyretic, anti-inflammatory, and analgesic agents, NSAIDs prove beneficial in addressing conditions such as muscle pain, dysmenorrhea, arthritis, pyrexia, gout, migraines, and serving as opioid-sparing agents in certain acute trauma cases [41,42]. However, the use of NSAIDs is associated with well-known adverse effects. In patients with renal dysfunction, the diminished prostaglandins via NSAIDs can lead to renal complications and electrolyte imbalance [43]. Potential cardiovascular adverse effects include myocardial infarction, thromboembolic events, and atrial fibrillation [44]. NSAIDs are not recommended in patients with hypersensitivity or salicylate hypersensitivity, previous allergic reactions, a history of coronary artery bypass graft surgery, and pregnant women during the third trimester [45].

Caffeine

Caffeine is an alkaloid and acts as an adenosine receptor blocker by competition mode, leading to a compensatory increase in adenosine. This subsequently stimulates circulating chemoreceptors and other receptors. This cascade results in heightened sympathetic tone, increased levels of catecholamines, peripheral vascular resistance, and augmented renin secretion. The net effect is an elevation in blood pressure [46]. At rest, caffeine is believed to enhance endothelial cell function by elevating intracellular calcium, prompting the expression of endothelial nitric oxide synthase, and thereby stimulating nitric oxide production [46]. Indirect tests in healthy individuals suggest improved endothelial cell function and vasodilation at rest. Therefore, adults engaging in daily activities while consuming this amount of caffeine are likely safe, given they are not caffeine-sensitive, pregnant, taking medications interacting with caffeine, or having medical conditions diminished by caffeine [47]. Various studies found minor changes in hemodynamic parameters, a slight increase in sympathetic activity, and subtle alterations in cardiac electrophysiological functions [48]. However, caution is advised for those consuming caffeine immediately before or during exercise, as it may potentially impede the typical physiological mechanisms supporting increased myocardial blood flow during heightened exercise requirements [47].

Monoamine Oxidase Inhibitors

Monoamine oxidase inhibitors (MAOIs) function by blocking the monoamine oxidase enzyme. This results in the breaking down of multiple neurotransmitters, such as tyramine, norepinephrine, serotonin, and dopamine, in the brain. By inhibiting the breakdown of these neurotransmitters, MAOIs elevate their levels, allowing them to persist and influence cells affected by depression [49]. Distinct from other antidepressants, MAOIs are effective in treating various forms of depression and addressing nervous system disorders such as panic disorder, social phobia, and depression with atypical features [50]. Additionally, MAOIs show benefits for patients with neurological disorders such as Parkinson’s disease and multiple system atrophy [51]. Common side effects include dry mouth, nausea, diarrhea, constipation, drowsiness, insomnia, dizziness, and lightheadedness. If administered via a patch, there is a possibility of a skin reaction at the patch site [52]. Individuals with a history of seizures or epilepsy, alcoholism, angina, severe headaches, blood vessel disease, diabetes, kidney or liver disease, recent heart attack or stroke, overactive thyroid, or pheochromocytoma should avoid MAOI therapy to prevent a hypertensive crisis [53]. MAOIs carry the risk of drug-to-drug interactions, drug-food interactions, and overdoses. Patients need to be cautious, avoiding combinations with other antidepressants such as selective serotonin reuptake inhibitors [54]. The findings are summarized in Table 4.

**Table 4:** Adjunctive agents used in the management of hypotension.

Medication, author, year, country	Study population	Mechanism of action	Benefits	Adverse effects/Other comments
Non-steroidal anti-inflammatory drugs: Vane, 1971, United States [40]	Electronic literature review	Inhibit cyclooxygenase, which increases vasoconstriction	Treats muscle pain, dysmenorrhea, arthritic conditions, and migraines	Adverse effects on the renal and cardiovascular systems. Patients can experience allergic reactions
Caffeine: Echeverri et al., 2010, England [46]	Electronic literature review	Increases adenosine, which increases peripheral vascular resistance	Increases blood pressure and improves endothelial cell function	Minor alterations in heart rate and blood pressure accompanied by an elevation in sympathetic activity. Considered safe for patients unless they are reactive to caffeine, pregnant, or other drugs that are not compatible with it
Monoamine oxidase inhibitors: Baker et al., 1992, United States [49]	Electronic literature review	Block monoamine oxidase enzyme inhibits the breakdown of neurotransmitters	Treats depression as well as other nervous system disorders	Mouth dryness, nausea, constipation, diarrhea, and sleeplessness are some of the side effects. Monoamine oxidase inhibitors may cause a hypertensive crisis

## Conclusions

This review underscores the diversity of drugs targeting different mechanisms to address hypotension, emphasizing their effectiveness in managing symptoms as well as highlighting associated risks, including adverse effects and potential drug interactions. The prescription of these medications should consider individual patient characteristics and underlying conditions. Ongoing monitoring of patients on hypotension drugs is crucial for identifying and managing potential adverse effects or interactions. This analysis concludes by emphasizing the importance of personalized treatment approaches and expressing optimism about advancing medical research for a more comprehensive understanding of hypotension and improved patient outcomes. The need for further research is emphasized to enhance understanding, safety assessment, and establish evidence-based guidelines through clinical trials across diverse patient populations. For example, future studies could examine the effectiveness of combining pharmacological and non-pharmacological interventions for a synergistic approach to managing orthostatic hypertension. Additionally, another study could assess the long-term effects of various treatments on patient outcomes, including cardiovascular events, quality of life, and overall mortality. Lastly, future studies could assess the feasibility and effectiveness of personalized treatment plans based on patient characteristics, including age, comorbidities, and the underlying cause of orthostatic hypertension.
